# Soybean and avocado unsaponifiables: a review of their potential use in the treatment of osteoarthritis

**DOI:** 10.3389/fvets.2024.1473688

**Published:** 2025-01-15

**Authors:** Ana Sabucedo-Suárez, Mónica López-Peña, María Permuy, Fernando Muñóz

**Affiliations:** ^1^Departamento de Anatomía, Producción Animal e Ciencias Clínicas Veterinarias, Facultade de Veterinaria, Campus Terra, Universidade de Santiago de Compostela, Lugo, Spain; ^2^IboneLab S.L., Laboratory of Biomaterials, Lugo, Spain

**Keywords:** osteoarthritis, OA, cartilage, soybean, unsaponifiables, ASU

## Abstract

Recent research has shown that Avocado-Soybean Unsaponifiables (ASU) greatly reduce the symptoms of osteoarthritis (OA). It’s yet unknown exactly how ASU works, however, it has been demonstrated to have analgesic and anti-inflammatory effects. These qualities can potentially lessen the need for non-steroidal anti-inflammatory medicines (NSAIDs) and their secondary effects. This review aims to examine the current literature on ASU, focusing on their efficacy, mechanism of action, and potential utility in treating OA for managing chronic pain associated with this condition. The literature review was conducted manually through Pubmed, Scopus and Web of Science (WOS) databases, covering studies from 2000 to 2022 with terms like “osteoarthritis,” “OA,” “animal models,” “ASU,” and “soy/avocado.” Two reviewers independently screened each article using inclusion and exclusion criteria and categorized the studies into *in vitro*, preclinical, and clinical groups. According to in vitro research, ASU affect the regulation of molecules related to OA, increasing structural elements like collagen and aggrecan and decreasing pro-inflammatory mediators. Although results vary, pre-clinical research in different animal models has demonstrated positive effects, such as ameliorating histopathological changes and reduced inflammation. Despite some discrepancies regarding structural changes in the joints, clinical trials typically demonstrate symptom relief and slow down the disease progression. While ASU demonstrates significant promise in alleviating OA symptoms and reducing reliance on NSAIDs, further research is essential to fully understand its mechanisms of action. More studies are needed to determine the precise pathways through which ASU exerts its effects and to establish the most effective dosages for its administration, either alone or in combination with other treatments.

## 1 Introduction

Osteoarthritis (OA) is an inflammatory disorder characterized by chronic progression. In humans, it has been reported that approximately 15% of the total population and more than 50% of the population over 60 years of age may suffer from OA at some point in their lives. In companion animals, it is one of the most common diseases, however, prevalence data are unclear, and the results of some authors contradict each other (1), estimating annual period prevalence of appendicular osteoarthritis in 2.5% (2). It is usually found in weight-bearing joints such as the hips and knees, adding to the belief that mechanical stress plays a major role in OA (3). It can be caused by trauma, metabolic, infectious or nutritional disorders, or congenital diseases affecting young individuals. Joint dysplasia, osteochondrosis dissecans, ununited ankle process, and patellar dislocation are examples of disorders that can lead to secondary OA (1).

The Osteo Arthritis Research Society International (OARSI) defines it as a disorder affecting mobile joints, characterized by cellular stress and extracellular matrix (ECM) degradation. This disorder involves different mechanisms such as articular cartilage damage, bone remodeling, new bone formation, synovial inflammation, and fibrosis of ligaments, tendons, menisci, and capsules (4); following an order which molecular level mechanisms are affected first, followed by anatomical and physiological disorders. These micro- and macro-injuries will result in maladaptive repair responses, causing the body’s attempts to compensate for the damage to result in a worsening of the process.

**Figure 1:**
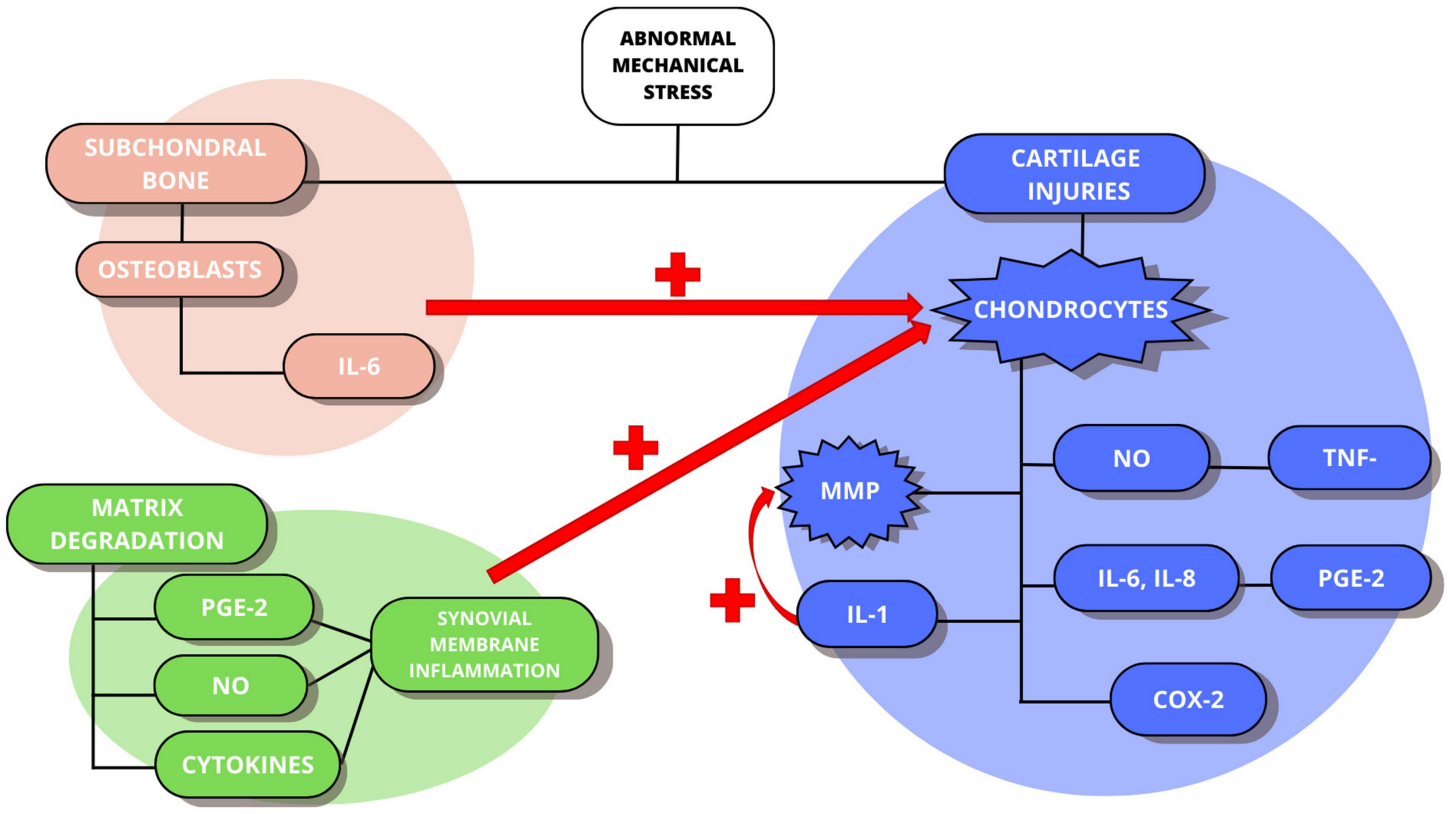
The maladaptive responses of the pathophysiology of OA. In blue the cartilage-cartilage loop. In orange the cartilage-subchondral bone loop. In green the cartilage-synovial membrane loop.

These maladaptive responses can be described as three bubbles or vicious circles involved in the pathophysiology of OA (5) and in the evolution of the process (Figure 1). Each of these three loops affects a specific structure of the joint architecture: cartilage, subchondral bone, and synovial membrane, respectively.

The first step is the presence of abnormal mechanical joint stress which will cause cartilage lesions and in turn, the activation of chondrocytes. Consequently, levels of proinflammatory cytokines such as interleukins (IL-1, IL-6, IL-8) and tumor necrosis factor (TNF-*α*) increase, releasing catabolic factors such as nitric oxide (NO), matrix metalloproteinases (MMPs), collagenases, aggrecanases, prostaglandin E2 (PGE2) and cyclooxygenase 2 (COX-2). In addition, IL-1 will play a major role in stimulating chondrocytes to produce more MMPs, promoting the continuation of matrix degradation. This corresponds to the first loop: cartilage-cartilage. At the same time, the mechanical stress will stimulate the osteoblasts present in the subchondral bone, which will release IL-6, also helping to stimulate the chondrocytes to produce more MMPs. This constitutes the second loop: cartilage-subchondral bone, leading to the appearance of fibrillation and erosion of the articular surface. Finally, as a product of matrix degradation, pro-inflammatory mediators (PGE-2, NO, or cytokines) also appear, which promote inflammation of the synovial membrane, stimulating the activation of chondrocytes and forming the third loop: cartilage-synovial membrane.

Specifically, PGE2 stimulates the production of degradative enzymes and inhibits the synthesis of cartilage components, particularly proteoglycans, and perpetuates the inflammatory response and cartilage damage by inducing the production of other pro-inflammatory mediators.

As the process evolves and in the later stages of OA, macroscopic lesions will become increasingly evident: erosions will occur throughout the cartilage reaching the subchondral bone, chondrocyte hypertrophy and clustering will lead to calcium deposition and osteophyte formation, catabolic factors will eventually lead to chondrocyte apoptosis, and all of this will eventually lead to sclerosis of the subchondral bone (6). In either case, it is a cause of reduced quality of life and its management can be complicated (7). It is known that in humans’ osteoarthritis can be extremely painful and in animals, it is no different. Several studies have shown that the positive response to analgesic therapy (versus other factors) may indicate that, for example, dogs experience a similar level of pain as humans (2).

Current strategies to combat OA are aimed at relieving pain, improving function and quality of life, and slowing down the process (8). Unfortunately, currently available medications cause numerous side effects and, in addition, a high number of patients do not achieve sufficient pain relief even with the combination of several analgesics (9).

The American College of Rheumatology (ACR) Arthritis Foundation and the Osteoarthritis Society International (OARSI) in their guidelines both agree in strongly recommending exercise, weight loss and, cognitive behavioral therapy from a physical approach. On the other hand, topical and oral NSAIDs are also strongly recommended in patients with knee OA (10).

However, for the use of oral NSAIDs, they are recommended but only in the absence of comorbid conditions and, if possible, with the addition of a proton pump inhibitor or selective COX-2 inhibitors (11). Therefore, research into new formulations is needed to clarify the mechanisms of pain and open new opportunities for targeted and more effective treatments.

Herbal medicines have a longstanding tradition in treating osteoarthritis, and recent research highlights their potential to alleviate pain and inflammation, through the interaction with inflammatory mediators and cartilage destruction, however, the mechanism of action of these is not yet fully understood (12). Despite this, evidence of their effectiveness in several studies provides a strong rationale for using them to treat OA symptoms (13). Related to this, it is important to note how other natural products, such as cannabidiol (CBD), are gaining popularity (14). The therapeutic use of CBD as an anti-inflammatory and immunomodulatory agent to treat chronic pain in both horses (15), and dogs is showing promising results, and there are already several studies describing pain relief related to OA (16). Therefore, herbal anti-inflammatory drugs provide a broad-spectrum mechanism of action. They interact to varying degrees in the inflammatory cascade, but experimental data also indicate interaction with the production of mediators of cartilage destruction. Other mechanisms of action include inhibition of elastase or hyaluronidase or antioxidant efficacy (17).

More specifically, avocado-soybean unsaponifiables (ASU) are made from avocado and soybean oil extracts in a 1:2 ratio and are among the slow-acting anti-inflammatory drugs (18). Its main components are the phytosterols beta-sitosterol, campesterol, and stigmasterol (19).

Many studies agree that ASU has an important role in inhibiting MMP activity and release (20), in its function as a chondroprotector by stimulating the synthesis of matrix components and promoting cartilage repair (1), decreasing the production of inflammatory cytokines (21) and increasing the production of collagen and aggrecan. Other authors suggest that ASU may act on osteoblasts present in the subchondral bone favoring the cartilage repair (22). However, most studies focus on various nutraceuticals and dietary supplements including ASU. They describe symptom improvement, but none focus on investigating structural changes in OA following treatment (23–26). Others, however, while agreeing that ASU treatment has a positive impact on symptomatology, do not observe significant changes in cartilage architecture (27).

In *in vitro* studies the model generally used to test ASU is based on reducing and controlling the activity of agents involved in the pathophysiology of OA by degrading cartilage and promoting tissue inflammation (28–32). In these studies, targeted IL-1 and IL-6 (21) inducing MMP activity, TNF-*α*, and the increased secretion of TGF-β1 and TGF-β2 (29, 30). Au et al. (33) found that ASU blocks the activation of COX-2 transcripts and lowers PGE2 secretion to basal levels.

Therefore, ASU has demonstrated chondroprotective, anabolic, and anticatabolic effects. It can promote the synthesis of extracellular matrix components (including collagen and aggrecan) in chondrocytes while suppressing the production of pro-inflammatory cytokines (IL-1β, IL-6, IL-8), PGE2, NO, and matrix metalloproteinases like MMP-13 (24, 33). These promote tissue maintenance and regeneration while reducing cartilage degeneration. Additionally, by blocking nitric oxide synthase (iNOS) and MMP-13 activity, ASU protects subchondral bone, halting bone resorption, and preserving bone density (18).

On a clinical level, oral administration of ASU has been shown to alleviate OA symptoms by preventing cartilage destruction and reducing pain and stiffness by improving the function of the affected joint (34). Therefore, it makes it possible to reduce the dependence and use of common analgesics (35).

Ultimately, although both anti-inflammatory and chondroprotective properties are described in a wide variety of studies, further studies are needed to support the efficacy of ASU. Furthermore, data on the concentrations of ASU components achieved in the blood (36) and on the most effective and efficient doses of ASU are unfortunately lacking.

The aim of this article is therefore to review the literature published so far on ASUs, their efficacy, their routes of action, and their potential effect in treating OA.

## 2 Materials and methods

The literature included in this review was conducted manually between September and December 2022.

### 2.1 Search strategy

The search for this review was performed through the following online scientific databases: Pubmed, Scopus and Web of Science (WOS). This search included studies published from 2000 to 2022.

The different publications were searched and identified, based on different search strategies, by permutations of the following terms: “osteoarthritis,” “OA,” “animal models,” “ASU” and/or “soy/avocado” as keywords.

We also included all articles found in the cross-references and bibliographies seen to be helpful or relevant.

### 2.2 Inclusion and exclusion criteria

#### 2.2.1 Inclusion criteria

- Articles in English.
- The presence of OA or simulation of its conditions*.
- Use of ASU as a substance to test its effect alone or in combination with other substances.
- *In vitro* studies.
- Preclinical studies in different animal species.
- Clinical studies in human and veterinary medicine.
- Chemical or surgical OA induction**.

*The study developed by de Paula et al. (37), which deals with Rheumatoid Arthritis (RA) rather than OA, was also included. The reasons for this are based on the common objective in both pathologies to investigate whether the effects of ASU include pain relief and slowing the progression of the pathology.

**As an exception, the article by Goudarzi et al. (30), in which a chemical induction of oedema was performed instead of OA, was also included. However, the study is directed toward OA and related to the presence of oedema in this pathology.

#### 2.2.2 Exclusion criteria

- Reviews.
- Abstracts and book chapters.
- Articles in a language other than English.
- Studies using substances other than ASU.
- Studies where the dose used of ASU is not specified.

### 2.3 Screening method and data extraction

The screening was carried out manually in two stages. First the titles and abstracts of each article were selected from the search results of the different databases. Then, duplicates were removed and two reviewers (A.S. and F.M.) independently screened them using the inclusion and exclusion criteria.

The same two reviewers (A.S. and F.M.) reviewed the full text of the articles for eligibility. For each study, relevant data were examined and extracted by dividing them into three distinct groups: *in vitro* studies, preclinical studies and clinical studies. Within the latter, they were subdivided into clinical studies conducted in humans and animals.

**Figure 2:**
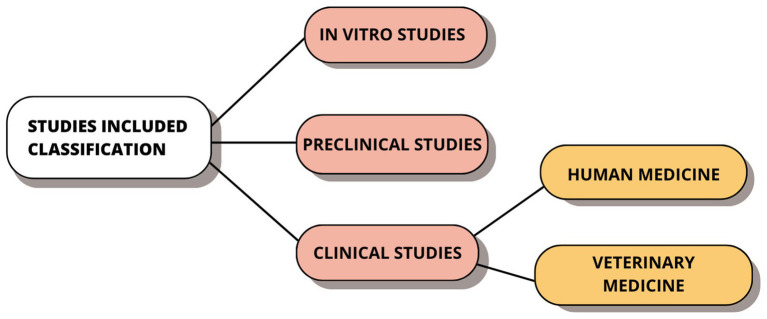
Classification of the studies included in this review into their three corresponding.

The materials and methods were later analyzed in depth, focusing on the species used, the type of induction, treatment used, and the results obtained for each article (Figure 2).

To conclude, the final selection of the studies included in this review was based on the inclusion and exclusion criteria explained above.

## 3 Results

**Figure 3:**
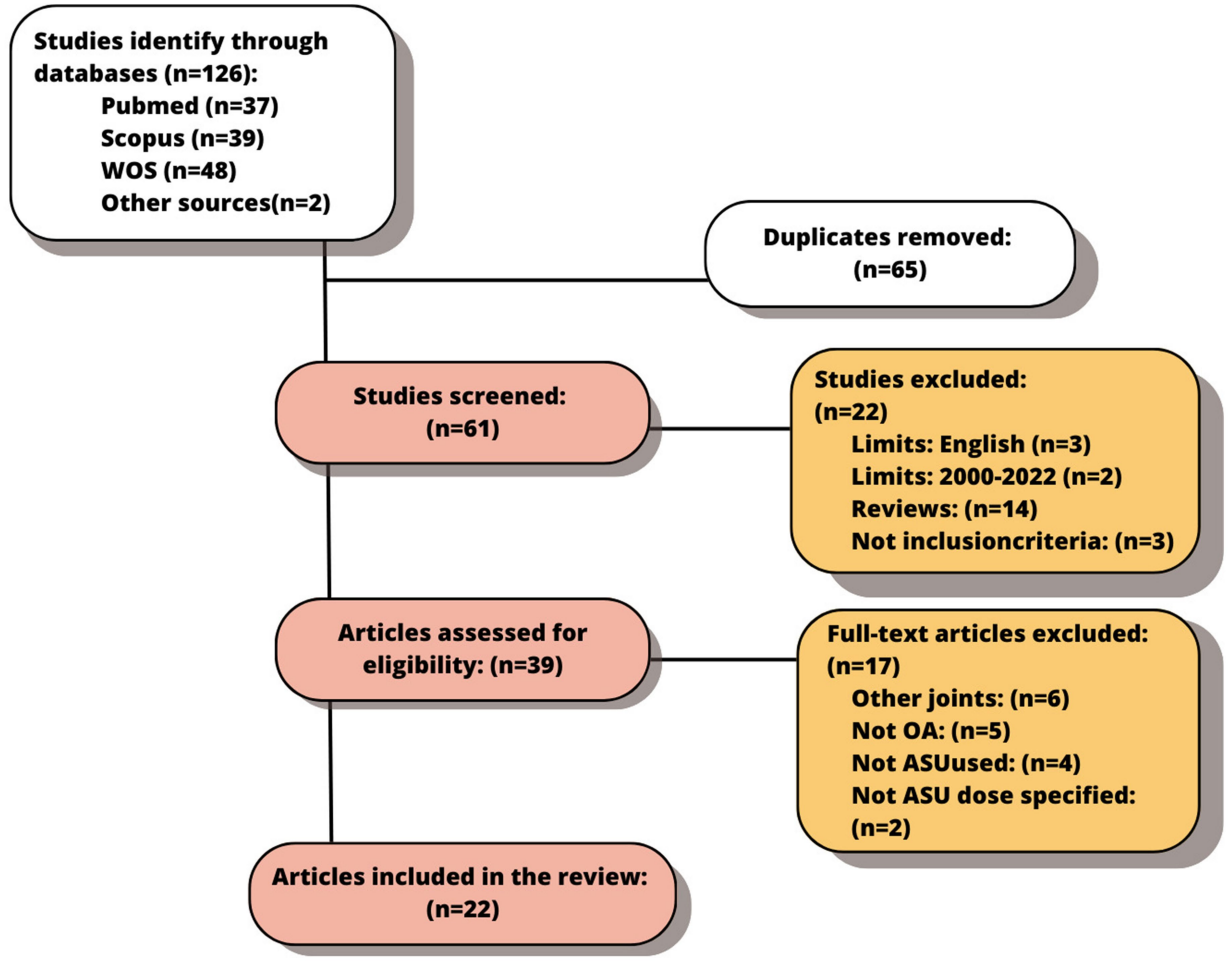
Flow chart of the final selected studies.

The initial search yielded 126 articles from the different databases. Once the duplicates were removed, the remaining 65 were screened. The title and abstract were reviewed, and the exclusion/inclusion criteria mentioned above were applied. A total of 22 articles were excluded at this stage. Finally, the full-text analysis of 39 studies was carried out of which, 22 articles were selected for this review (Figure 3).

### 3.1 *In vitro* studies

A summary of the tests included in this section can be found in Table 1. They are explained in more detail below.

**Table 1:** Summary of the characteristics of the *in vitro* studies included.

*In vitro* studies			
References	Material and methods	Treatment (concentrations + time of incubation)	Results
Henrotin et al. (32)	Model of isolated OA chondrocytes + IL-1β	10 μg/mL ASU + 15 days	ASU promoted the recovery of aggrecan synthesis after 3 days of IL-1β treatment. It inhibited the basal production of MMP-3, IL-6, IL-8, NO and PGE2.
Au et al. (33)	Monocyte/macrophage-like cell model Biomarkers: IL-1β, TNF-*α*, COX-2 and iNOS	25 μg/mL ASU + 3 days	ASU suppresses gene expression of potent pro-inflammatory cytokines
Gabay et al. (28)	Mouse/human chondrocytes + IL-1β + mechanical stress with/without ASU	10 μg/mL ASU + 2 days	ASU decreases MMP-3 and MMP-13 expression and PGE2 release
Lippiello et al. (19)	Model of cow metacarpal joint chondrocytes	30 μg/mL ASU + 1 day 75 μg/mL ASU + 3 days	The results suggest a relationship between ASU content and biological activity
Grzanna et al. (38)	Monocyte/macrophage-like cell model Combination of ASU + CS	8,3 μg/mL ASU + 1 day	ASU and CS reduces TNF-α expression but does not block cytokine expression
Ownby et al. (39)	Model of equine chondrocytes pre-incubated with ASU + EGCG + IL-1β and TNF-α	8,3 μg/mL ASU 0,04 μg/mL EGCG + 1 day	ASU + EGCG activity affects a wide range of inflammatory molecules in addition to decreasing PGE2 synthesis in activated chondrocytes
Frondoza et al. (40)	Model of equine chondrocytes Combination of ASU + LA	8,3 μg/mL ASU 2,5 μg/mL LA + 1 day	LA: inhibits IL-1β and H_2_O_2_ ASU: inhibits IL-1β activity, but not H_2_O_2_ ASU + LA: inhibits PGE2 production
Grzanna et al. (41)	Model of chondrocyte microcarriers + IL-1β Biomarkers: PGE2, IL-6, IL-8 and MCP-1 Combination of carprofen (ASU + GLU + CS)	8,3 μg/mL ASU 11 μg/mL GLU 20 μg/mL CS + 1 day	Carprofen + (ASU + GLU + CS): inhibits PGE2 production more than the agents alone
Teimourinejad et al. (42)	hADSC model in fibrin Combination of PFE + ASU	10 μg/mL ASU 10 μg/mL PFE 5 μg/mL ASU + 5 μg/mL PFE + 14 days	PFE + ASU produced a smaller effect than PFE alone, but larger than ASU

Of the nine articles included in this section, Henrotin et al. (32), Au et al. (33), and Gabay et al. (28) share very similar lines of research. These three studies use human chondrocytes stimulated or not with IL-1β. In the case of Au et al. (33) they also tested multiple cell types involved in joint inflammation, i.e., monocytes/macrophages and fibroblasts. As biomarkers, they used a wide range, of IL-1β, TNF-*α*, COX-2, and iNOS, PGE2, and NO. Henrotin et al. (32) and Gabay et al. (28) tested the efficacy of ASU on MMP-3 and -13 and PGE2 expressions. As for the results, they showed that ASU suppresses gene expression of potent proinflammatory cytokines, including TNF-a, IL-1b, COX-2, and iNOS, as well as MMP-3 and -13 and PGE2 release. Notably, Au et al. (33) showed for the first time that the anti-inflammatory effects of ASU are not limited to chondrocytes and fibroblasts but extend to surrogate cells, and monocytes/macrophages.

A similar study to the previous ones was designed by Lippiello et al. (19), to clarify whether the sterol content in ASU could play a role in the biological activity of articular chondrocytes. Different analytical tools were used, for samples with ASU and before culture with bovine chondrocytes, to observe the variation in sterol content. Anabolic and anti-inflammatory activity was then analyzed, suggesting a dose- and time-dependent relationship between ASU content and chondrocyte biological activity.

On the other hand, the articles by Grzanna et al. (38), Ownby et al. (39), Frondoza et al. (40), Grzanna et al. (41), and Teimourinejad et al. (42) use different supplements in combination with ASUs to test whether their effect is greater together or alone. These supplements include chondroitin sulfate (CS) (38), epigallocatechin gallate (EGCG) (26), *α*-lipoic acid (LA) (40), or pomegranate extract (PFE) (42). In addition, the article by Grzanna et al. (41) used a combination of ASU, glucosamine (GLU), and chondroitin sulfate (CS) with Carprofen. These studies showed that ASU decreases various types of cytokines expression, and PGE2 synthesis in chondrocytes, and inhibits H_2_O_2_.

To sum up, all articles reported that the combination of ASU with other supplements has more effect than alone. Therefore, the main objective should be to reduce or inhibit the expression of all these molecules without blocking the expression of cytokines, enhancing the potential to attenuate inflammation without reducing cytokines below the levels necessary for normal physiological function. In other words, these combinations could be used as an alternative or complement to conventional pharmacological treatments for the treatment of OA and could reduce the dose of NSAIDs and their side effects.

### 3.2 Preclinical studies

A summary of the studies included in this section can be found in Table 2. However, they are explained in more detail below.

**Table 2:** Summary of the characteristics of the preclinical studies included.

Preclinical studies					
References	Study groups	Study size (adults)	Diagnosis/induction	Treatment (orally administrated)	Results
Cake et al. (43)	MenX+ASU: 8 MenX+Placebo:8 NOC:8	48 sheeps	Surgical induction: bilateral lateral meniscectomy	900 mg/working day ASU For 3 or 6 months	Partial preservation of cartilage integrity in ASU-treated individuals versus the placebo and NOC groups
Altinel et al. (46)	Control:8 ASU high dose: 8 ASU low dose: 8	24 dogs	Knee joint fluid sampling post-treatment	300 mg/day ASU or 300 mg/3 times a day ASU For 3 months	ASU-treated groups show increases levels of TGF-β1 and TGF-β2 in joint fluid. However, TGF levels were not significantly altered by the different dosages
Kawcak et al. (44)	Placebo group: 8 ASU group:8	16 horses	Surgical induction: osteochondral fragmentation	300 mg/day ASU or placebo For 70 days	ASU group did not show a reduction in clinical signs compared with the placebo group
Boileau et al. (45)	Placebo group: 8 ASU group:8	16 dogs	Surgical induction: anterior cruciate ligament section	200–250 mg/day ASU* or placebo For 8 weeks	The severity of the lesions was significantly decreased in the ASU-treated group compared with the placebo group
Al-Alfify et al. (18)	Control: 10 MIA:10 MIA + ASU:10	30 rats	Chemical induction of OA: intraarticular injection of MIA	5,5–6,9 mg/day ASU* For 3 weeks	ASU maintained matrix staining, decreased cellular abnormalities, and stopped cartilage structural degradation induced by MIA
de Paula et al. (37)	Control: 30 ASU:30 Arthritis: 30 Arthritis+ASU: 30	120 rats	Surgical induction: Systemic immunization + implant placement	180–198 mg/day ASU* For 15, 30 or 60 days	The ASU-treated group showed an improvement in osseointegration compared with the arthritic group
Goudarzi et al. (30)	Control:6 ASU:6 Nanopliposome:6 NANOCEN:6 Ibuprofen: 6	30 mice	Paw inflammation	Topical administration** 0,4 mg/day ASU For 30 days	NANOCEN showed robust anti-inflammatory and analgesic effect superior to ibuprofen

*These dosages vary according to the weight of each animal.

**All the studies administrated ASU orally except for Goudarzi et al. (30).

To investigate the effect of oral ASU, Cake et al. (43) and Kawcak et al. (44) conducted their studies in sheep and horses respectively, inducing OA chirurgically. Both studies divided the animals into two groups: one treated with placebo and the other with ASU. In the case of Cake et al. (43) they used a dose of 900 mg/day of ASU significantly higher than that used in the study by Kawcak et al. (44) 300 mg/day of ASU. In terms of clinical signs, Kawcak et al. (44) observed that these did not disappear with ASU treatment, but by macroscopic examination, they were able to conclude that it did reduce the severity of articular cartilage erosion and synovial hemorrhage. Cake et al. (43) performed histomorphometry tests on histological sections measuring the areas of total cartilage, non-calcified cartilage, and subchondral bone thickness, as well as the intensity of toluidine blue. However, they failed to demonstrate any significant effect of ASU treatment. However, imaging analysis demonstrated that cartilage integrity was partially preserved in ASU-treated individuals (although other causes could not be excluded).

On the other hand, Boileau et al. (45) and Al-Afify et al. (18), used immunohistochemical techniques to evaluate the expression of MMP-13 and iNOS (nitric oxide synthase). Both articles revealed a significant reduction in the level of iNOS and MMP-13 in the ASU-treated groups. This finding is consistent with other studies describing NO as one of the important mediators of articular cartilage and subchondral bone damage. It was also found to modulate the activity of MMP-13. MMPs and other catabolic enzymes, particularly MMP-1 and 13, mediate cartilage collagen degradation during OA. In terms of histological evaluation, both studies showed a reduction in lesion severity and a decrease in subchondral bone volume loss (45), and articular cartilage showed increased cellularity and a higher degree of matrix staining (18).

It is important to highlight that Altinel et al. (46) conducted a different study by dividing the 24 dogs in the study into three groups: a control group (with a normal diet), a group treated with high doses of ASU (300 mg/day) and another group treated with low doses (300 mg/3 days). They analyzed TGF-*β* levels in joint fluid samples. They concluded that ASU treatment does, indeed, increase TGF-β1 and TGF-β2 levels in joint fluid for 3 months, but further studies are needed to define the mechanism of the symptomatic effects of ASU treatment on osteoarthritic joints.

In 2018, the study by de Paula et al. (37) evaluated osseointegration *in vivo* (120 rats) in an experimental model of rheumatoid arthritis. Previous studies have shown that the usefulness of ASU in pathologies in which the formation of new bone tissue is beneficial is subtle. However, this study proposes that their use could have a greater impact on individuals with altered bone tissue metabolism. The results showed a higher osseointegration potential in the ASU-treated groups (180–198 mg/day ASU for 15, 30 or 60 days). The study comments that this finding may be because, with the anti-inflammatory potential of ASU, bone resorption around the implant is reduced by stimulating connective tissue proliferation. Increased expression of TGF-β1 was also observed, indicating increased connective tissue proliferation and coinciding with the findings of Goudarzi et al. (30) and Głuszko and Stasiek (29). It can be concluded from this study that treatment with the ASU improved osseointegration, particularly in animals with induced arthritis.

Finally, Goudarzi et al. (30) carried out a topical study conducted in mice to formulate nanoliposomes that facilitate the cutaneous administration (NANOCEN) of ASU (0.4 mg/day for 30 days) and then evaluate its anti-inflammatory and analgesic effects in comparison with 5% ibuprofen gel. The results revealed effective inhibition of inflammation and even better pain relief than ibuprofen 5%.

### 3.3 Clinical studies

The summary of the tests included in this section can be found in Table 3.

**Table 3:** Summary of the characteristics of the clinical studies included.

Clinical studies					
References	Study groups	Study size	Diagnosis	Treatment (Orally administrated)	Results
Human clinical trials					
Appelboom and Schuerman (47)	ASU300: 86 ASU600: 86 Placebo: 86	260 patients (45–80 years)	Femoro-tibial OA	300 mg/day ASU 600 mg/day ASU Or placebo For 3 months	Significant differences between the ASU 300 and 600 groups versus the placebo group. No significant differences between the 300 and 600 ASU groups
Lequesne et al. (49)	ASU: 54 Placebo: 54	108 patients	Hip OA	300 mg/day ASU Or placebo For 2 years	No structural effect
Pavelka et al. (48)	ASU 300:142 CS 400: 121	263 patients (45 or > 45 years)	Femoro-tibial OA	300 mg/day ASU 400 mg 3 times/day CS For 6 months	No difference between CS and ASU
Maheu et al. (25)	Placebo: 179 ASU:166	345 patients	Hip OA	300 mg/day ASU Or placebo For 3 years	No significant differences between the two groups. But slower progression in the ASU group.
Głuszko and Stasiek (29)	Single group	4,186 patients (average age 60 years)	Knee OA	300 mg/day ASU For 6 months	ASU achieves significant clinical improvement
Jangravi et al. (34)	PD: 20 DP: 20	40 patients (<60 years)	Bilateral knee OA	300 mg/day ASU PD: placebo 3 months + ASU 3 months DP: ASU 3 months + placebo 3 months	ASU is effective as an antioxidant
Veterinary clinical trials					
Kwananocha et al. (50)	Single group	10 dogs (adults)	Hip OA	900 mg/day GLU 350 mg/day CS 900 mg/day ASU For 4 weeks	Inconclusive. Possible insufficient dose.

#### 3.3.1 Human clinical trials

The seven articles included in this section evaluated the function of ASUs in the knee or hip but in different ways.

Appelboom and Schuerman (47), Pavelka et al. (48), and Głuszko and Stasiek (29) evaluated symptomatic effects in different groups of patients. In the case of Appelboom and Schuerman (47), they separated patients into three groups: a control group, one treated with 300 mg/day ASU, and another with 600 mg/day ASU for 3 months. Pavelka et al. (48), on the other hand, compared the effects of ASU (300 mg/day) with those of CS (400 mg/day). Despite their differences, the results of these three articles are similar: they proved the efficacy of ASU in relieving symptoms. However, Appelboom and Schuerman (47) and Pavelka et al. (48) find no differences between the different doses of ASU used or between ASU and CS, respectively. Of note, Appelboom and Schuerman (47) also found that 71% of patients had reduced their NSAIDs intake by more than 50%, proving that ASU can help to reduce the side effects of painkillers by reducing their consumption.

Structural joint effects were analyzed by Lequesne et al. (49) and Maheu et al. (25) in the hip joint in both cases. The dose used in these studies is 300 mg/day for 2 years or 6 months. Lequesne et al. (49) used the joint space width as the main criteria, and could not demonstrate any structural effect, however, the results suggested that ASU breaks joint space loss. Maheu et al. (25) also used radiographs and clinical observations, they also showed that ASU reduced the progression of joint space loss and concluded that ASU may have a role as a modifier of joint structure in OA.

Unlike the other studies, Jangravi et al. (34) evaluated the effects of ASU on oxidative stress and serum antioxidant levels in two groups of patients, those who were treated with placebo and then with ASU and those who were treated first with ASU and then with placebo. Both groups were given 300 mg/day of ASU for 3 months. The results showed a decrease in the serum level of a marker of oxidative stress, significantly reduced levels of antioxidants after 3 months of treatment and demonstrated the independence of the order of treatment administration concerning the effect of the ASU.

#### 3.3.2 Veterinary clinical trials

In 2016, Kwananocha et al. (50) conducted a study on 40 dogs with a history of lameness that aimed to evaluate and compare the effectiveness of disease-modifying osteoarthritis agents (DMOAAs). For this purpose, 40 dogs were included in the study and assigned into 4 treatment groups (*n* = 10). In one of the groups, a combination of glucosamine-chondroitin sulfate (GC) and avocado/soybean unsaponifiables (ASU) were used. The combination GC-ASU contained glucosamine 900 mg, chondroitin sulfate 350 mg and ASU 90 mg. Finally, after 4 weeks of treatment, the results of the study were inconclusive. However, based on the literature and the results of other preclinical studies in which the efficacy of ASU was proven, they justify this result by a possible insufficient dose of the combination of GC.

## 4 Discussion

### 4.1 *In vitro* studies

All included articles used isolated chondrocytes or similar cells such as monocytes or macrophages, but not all enriched the culture medium with IL-1β. Three of them do not use IL-1β as activation in contrast to the rest of the literature. Henrotin et al. (32), however, unites the two approaches and tests in the same study, the culture using IL-1β on the one hand and without using it on the other.

Another difference observed is the combination or not of ASU with other substances. Some supplemented ASU with LA others with PFE and, finally, one study tried combining them with CS.

Most of the studies agree in selecting common biomarkers: MMP-13, PGE2, and different ILs with some exceptions. Three articles also measure TNF-*α*, others also measure NO and three more deviate and measure ECGC and H_2_O_2_, respectively. In addition, Au et al. (33) also add the measurement of COX-2 and iNOS.

On the other hand, they all use different methods to get the results, ranging from immunoassays, electrophoresis, immunotransfer or immunohistochemistry; to spectrometry and chromatography, rPCR, and Western blotting. However, the results are similar in all articles. In one way or another, they all agree on either a decrease in proinflammatory molecules or an increase in structural molecules, such as aggrecan or collagen, and reach the same conclusion by different routes and methods. The 7 *in vitro* studies included in this review claim that there is clear evidence that ASU affects the regulation of these molecules and, therefore, sufficient reasons to test its efficacy in preclinical studies.

### 4.2 Preclinical studies

The first difference observed in the preclinical studies is the variety of species used. A total of 7 studies including in this review 2 used dogs, two rats, one horses, one sheep and, finally one conducted their study in mice.

It should be noted that most authors used surgical induction as a method to induce OA. However, they used different vias. For example, Cake et al. (43) performed a bilateral lateral meniscectomy, Kawcak et al. (44) osteochondral fragmentation at the carpal joint and Boileau et al. (45) sectioned the anterior cruciate ligament of the knee. Contrary to the rest of the literature, two studies did not use surgical but chemical induction. The first one used an intraarticular injection of MIA to induce OA and the other one injected carrageenan into the subplantar tissue of the right paw to induce edema.

On the other hand, we found no similarities between the different studies in the duration of the treatment, or the doses used. In this last case, they vary from 900 mg per day to 10 mg per kg and day, and if we look at the duration of treatment, from 70 days to 6 months, there is also a significant difference.

In terms of outcomes, there is also controversy. While some articles did not observe a decrease or disappearance of clinical signs, the rest of the authors describe the results as positive. Goudarzi et al. (30) revealed a decrease in inflammation and pain relief and Boileau et al. (45) an improvement of the histopathology of the lesion. Furthermore, and as an example of contradiction within the same study, although Cake et al. (43) found no significant differences in the macroscopic study and histology, once the statistics were performed, they did find significant differences.

To conclude, some of the authors point out the variation in biomarkers. Three of them agree on the increased expression of TGF-1β and two describe the decreased expression of both iNOS and MMP-13. However, all the publications agree on the beneficial effects of ASU on the OA.

### 4.3 Clinical studies

In contrast to the pre-clinical studies where there was a great deal of variety in the species included in the investigations, almost all the articles included in this review are studies conducted in human medicine. The exception is Kwananocha et al. (50), which is the only case of a clinical trial conducted on dogs and focused on veterinary medicine.

The same is true for the doses used. All the articles are unanimous in the use of 300 mg per day except Kwananocha et al. (50) who used 90 mg. In addition, Appelboom and Schuerman (47) formed two groups and administered 600 mg per day to the second group, although, in the end, the results found no difference between the two.

In terms of the joint affected, there are differences. Two of the studies focused on the femorotibial joint, two on the hip and the other two on the knee.

Finally, the results all agree that they indicate a slowing of the progression of the pathology and alleviation of symptoms. Although, it is true that both Lequesne et al. (49) and Maheu et al. (25), who studied structural effects, failed to prove any positive change after treatment. Importantly, Kwananocha et al. (50) cannot be compared on this point as their results were inconclusive due to a possibly insufficient dose.

## 5 Conclusion

Based on the literature found and consulted, we can affirm that most of the studies reach a common answer: ASU alone or in combination with other substances helps to a great extent to reduce the symptoms of OA, but without locating all the ways of action in which it participates. In other words, it produces an analgesic and anti-inflammatory effect capable of reducing the doses of NSAIDs and therefore their side effects, which are necessary to control the chronic pain caused by this pathology. However, as it acts on multiple types of cells involved in inflammation and other mechanisms not yet fully understood, further studies are needed to find out how it produces these beneficial effects and by which pathways, as well as to establish effective doses for the administration of ASU, alone or in combination with other drugs or supplements.

## References

[1] Henrotin Y Sanchez C Balligand M. Pharmaceutical and nutraceutical management of canine osteoarthritis: present and future perspectives. Vet J. (2005) 170:113–23. doi: 10.1016/j.tvjl.2004.08.014 15993795

[2] Anderson KL O’Neill DG Brodbelt DC Church DB Meeson RL Sargan D. Prevalence, duration and risk factors for appendicular osteoarthritis in a UK dog population un- der primary veterinary care. Sci Rep. (2018) 8:5641. doi: 10.1038/s41598-018-23940-zt 29618832 PMC5884849

[3] Taylor JF Goudarzi R Yazdi PG Pedersen BA. In vitro effects of arthrocen, an Avocado/soy unsaponifiables agent, on inflammation and global gene expression in human monocytes. IJC. (2017) 9:31. doi: 10.5539/ijc.v9n4p31 PMC5903287 29675116

[4] Goudarzi R Taylor JF Yazdi PG Pedersen BA. Effects of Arthrocen, an avocado/soy unsaponifiables agent, on inflammatory mediators and gene expression in human chondrocytes. FEBS Open Bio. (2017) 7:187–94. doi: 10.1002/2211-5463.12176 PMC5292663 28174685

[5] Edgard Henrotin Y. Avocado/soybean unsaponifiables (Piacledine^®^300) show beneficial effect on the metabolism of osteoarthritic cartilage, synovium and subchondral bone: an overview of the mechanisms. AIMS Med Sci. (2018) 5:33–52. doi: 10.3934/medsci.2018.1.33

[6] Primorac D Molnar V Rod E Jeleč Ž Čukelj F Matišić V. Knee osteoarthritis: a review of pathogenesis and state-of-the-art non-operative therapeutic considerations. Genes. (2020) 11:854. doi: 10.3390/genes11080854, PMID: 32722615 PMC7464436

[7] Rychel JK. Diagnosis and treatment of osteoarthritis. Top Companion Anim Med. (2010) 25:20–5. doi: 10.1053/j.tcam.2009.10.005 20188335

[8] American College of Rheumatology Subcommittee on Osteoarthritis Guidelines. Recommendations for the medical management of osteoarthritis of the hip and knee: 2000 update. Arthritis Rheum. (2000) 43:1905–15. doi: 10.1002/1529-0131(200009)43:9<1905::AID-ANR1>3.0.CO;2-P 11014340

[9] Feldman P Khanna R. Challenging the catechism of therapeutics for chronic neuropathic pain: targeting CaV2.2 interactions with CRMP2 peptides. Neurosci Lett. (2013) 557:27–36. doi: 10.1016/j.neulet.2013.06.057 23831344 PMC3849117

[10] Kolasinski SL Neogi T Hochberg MC Oatis C Guyatt G Block J. 2019 American College of Rheumatology/Arthritis Foundation guideline for the Management of Osteoarthritis of the hand, hip, and knee. Arthritis Care Res. (2020) 72:149–62. doi: 10.1002/acr.24131, PMID: 31908149 PMC11488261

[11] Bannuru RR Osani MC Vaysbrot EE Arden NK Bennell K Bierma-Zeinstra SMA. OARSI guidelines for the non-surgical management of knee, hip, and polyarticular osteoarthritis. Osteoarthr Cartil. (2019) 27:1578–89. doi: 10.1016/j.joca.2019.06.011, PMID: 31278997

[12] Cameron M Chrubasik S. Oral herbal therapies for treating osteoarthritis. Cochrane Database Syst Rev. (2014) 2014:CD002947. doi: 10.1002/14651858.CD002947.pub2 24848732 PMC4494689

[13] Cameron M Gagnier JJ Little CV Parsons TJ Blümle A Chrubasik S. Evidence of effectiveness of herbal medicinal products in the treatment of arthritis. Phytother Res. (2009) 23:1497–515. doi: 10.1002/ptr.3007 19856319

[14] Interlandi C Tabbì M di S D’Angelo F Costa GL Arfuso F. Improved quality of life and pain relief in mature horses with osteoarthritis after oral transmucosal cannabidiol oil administration as part of an analgesic regimen. Front Vet Sci. (2024) 11:1341396. doi: 10.3389/fvets.2024.1341396, PMID: 38379920 PMC10876772

[15] Sánchez-Aparicio P Florán B Rodríguez Velázquez D Ibancovichi JA Varela Guerrero JA Recillas S. Cannabinoids CB2 receptors, one new promising drug target for chronic and degenerative pain conditions in equine veterinary patients. J Equine Vet Sci. (2020) 85:102880. doi: 10.1016/j.jevs.2019.102880 31952645

[16] Gugliandolo E Licata P Peritore AF Siracusa R D’Amico R Cordaro M. Effect of Cannabidiol (CBD) on canine inflammatory response: an ex vivo study on LPS stimulated whole blood. Vet Sci. (2021) 8:185. doi: 10.3390/vetsci8090185, PMID: 34564578 PMC8473042

[17] Chrubasik JE Roufogalis BD Chrubasik S. Evidence of effectiveness of herbal anti- inflammatory drugs in the treatment of painful osteoarthritis and chronic low back pain. Phytother Res. (2007) 21:675–83. doi: 10.1002/ptr.2142 17444576

[18] Al-Afify ASA El-Akabawy G El-Sherif NM El-Safty FENA El-Habiby MM. Avocado soybean unsaponifiables ameliorates cartilage and subchondral bone degeneration in mono-iodoacetate-induced knee osteoarthritis in rats. Tissue Cell. (2018) 52:108–15. doi: 10.1016/j.tice.2018.05.001 29857819

[19] Lippiello L Nardo JV Harlan R Chiou T. Metabolic effects of avocado/soy unsaponifiables on articular chondrocytes. Evid Based Complement Alternative Med. (2008) 5:191–7. doi: 10.1093/ecam/nem132 PMC2396479 18604259

[20] Simental-Mendía M Sánchez-García A Acosta-Olivo CA Vilchez-Cavazos F Osuna-Garate J Peña-Martínez V. Efficacy and safety of avocado-soybean unsaponifiables for the treatment of hip and knee osteoarthritis: a systematic review and meta-analysis of randomized placebo-controlled trials. Int J Rheum Dis. (2019) 22:1607–15. doi: 10.1111/1756-185X.13658, PMID: 31328413

[21] Henrotin YE Labasse AH Jaspar JM de D Zheng SX Guillou GB. Ef- fects of three avocado/soybean unsaponifiable mixtures on metalloproteinases, cytokines and prostaglandin E2 production by human articular chondrocytes. Clin Rheumatol. (1998) 17:31–9. doi: 10.1007/BF01450955, PMID: 9586676

[22] DiNubile NA. A potential role for avocado- and soybean-based nutritional supplements in the Management of Osteoarthritis: a review. Phys Sportsmed. (2010) 38:71–81. doi: 10.3810/psm.2010.06.1785 20631466

[23] Ernst E. Avocado soybean unsaponifiables (ASU) for osteoarthritis? A systematic review. Clin Rheumatol. (2003) 22:285–8. doi: 10.1007/s10067-003-0731-4, PMID: 14576991

[24] Christensen R Bartels EM Astrup A Bliddal H. Symptomatic efficacy of avocado– soybean unsaponifiables (ASU) in osteoarthritis (OA) patients: a meta-analysis of randomized controlled trials. Osteoarthr Cartil. (2008) 16:399–408. doi: 10.1016/j.joca.2007.10.003 18042410

[25] Maheu E Cadet C Marty M Moyse D Kerloch I Coste P. Randomised, controlled trial of avocado–soybean unsaponifiable (Piascledine) effect on structure modification in hip osteoarthritis: the ERADIAS study. Ann Rheum Dis. (2014) 73:376–84. doi: 10.1136/annrheumdis-2012-202485, PMID: 23345601 PMC3913295

[26] Liu X Machado GC Eyles JP Ravi V Hunter DJ. Dietary supplements for treating osteoarthritis: a systematic review and meta-analysis. Br J Sports Med. (2018) 52:167–75. doi: 10.1136/bjsports-2016-097333 29018060

[27] Trifunovic-König M Klose P Cramer H Koch A Dobos G Langhorst J. Phytotherapy for osteoarthritis. Z Phytother. (2017) 37:242–7. doi: 10.1055/s-0042-121567

[28] Gabay O Gosset M Levy A Salvat C Sanchez C Pigenet A. Stress-induced signaling pathways in hyalin chondrocytes: inhibition by avocado–soybean unsaponifiables (ASU). Osteoarthr Cartil. (2008) 16:373–84. doi: 10.1016/j.joca.2007.06.016, PMID: 17707661

[29] Głuszko P Stasiek M. Symptom-modifying effects of oral avocado/soybean unsaponifiables in routine treatment of knee osteoarthritis in Poland. An open, prospective observational study of patients adherent to a 6-month treatment. Reumatologia. (2016) 5:217–26. doi: 10.5114/reum.2016.63661 PMC5149568 27994265

[30] Goudarzi R Amini S Dehpour AR Partoazar A. Estimation of anti-inflammatory and analgesic effects of topical NANOCEN (Nanoliposomal Arthrocen) on mice. AAPS PharmSciTech. (2019) 20:233. doi: 10.1208/s12249-019-1445-5 31236745

[31] Henrotin Y Lambert C Couchourel D Ripoll C Chiotelli E. Nutraceuticals: do they rep- resent a new era in the management of osteoarthritis? – a narrative review from the lessons taken with five products. Osteoarthr Cartil. (2011) 19:1–21. doi: 10.1016/j.joca.2010.10.017 21035558

[32] Henrotin YE Deberg MA Crielaard JM Piccardi N Msika P Sanchez C. Avocado/soybean unsaponifiables prevent the inhibitory effect of osteoarthritic subchondral osteoblasts on aggrecan and type II collagen synthesis by chondrocytes. J Rheumatol. (2006) 11:1668–1678. 16832844

[33] Au RY Al-Talib TK Au AY Phan PV Frondoza CG. Avocado soybean unsaponifiables (ASU) suppress TNF-α, IL-1β, COX-2, iNOS gene expression, and prostaglandin E2 and nitric oxide production in articular chondrocytes and monocyte/macrophages. Osteoarthritis Cartilage. (2007) 15:1249–55. doi: 10.1016/j.joca.2007.07.009 17845860

[34] Jangravi Z Basereh S Zaree Mahmoudabadi A Saberi M Alishiri GH Korani M. Avocado/soy unsaponifiables can redress the balance between serum antioxidant and oxidant levels in patients with osteoarthritis: a double-blind, randomized, placebo-controlled, crossover study. J Complement Integr Med. (2021) 18:769–74. doi: 10.1515/jcim-2020-0265 33794080

[35] Christiansen BA Bhatti S Goudarzi R Emami S. Management of Osteoarthritis with avocado/soybean Unsaponifiables. Cartilage. (2015) 6:30–44. doi: 10.1177/1947603514554992 25621100 PMC4303902

[36] Comblain F Serisier S Barthelemy N Balligand M Henrotin Y. Review of dietary sup- plements for the management of osteoarthritis in dogs in studies from 2004 to 2014. J Vet Pharmacol Therap. (2016) 39:1–15. doi: 10.1111/jvp.12251 26205697

[37] de Paula L de Oliveira G Pinotti F Grecchi B de Aquino S Marcantonio R. Effect of avocado/soybean Unsaponifiables (ASU) on Osseointegration in rats with experimental arthritis. Int J Oral Maxillofac Implants. (2018) 33:603–12. doi: 10.11607/jomi.6124, PMID: 29763498

[38] Grzanna MW Ownby SL Heinecke LF Au AY Frondoza CG. Inhibition of cytokine expression and prostaglandin E2 production in monocyte/macrophage-like cells by avocado/soybean unsaponifiables and chondroitin Sulfate. J Complement Integr Med. (2010) 7:10. doi: 10.2202/1553-3840.1338

[39] Ownby SL Fortuno LV Au AY Grzanna MW Rashmir-Raven AM Frondoza CG. Expression of pro-inflammatory mediators is inhibited by an avocado/soybean unsaponifiables and epigallocatechin gallate combination. J Inflamm. (2014) 11:8. doi: 10.1186/1476-9255-11-8 PMC3983882 24678847

[40] Frondoza CG Fortuno LV Grzanna MW Ownby SL Au AY Rashmir-Raven AM. α- Lipoic acid potentiates the anti-inflammatory activity of avocado/soybean unsaponifiables in chondrocyte cultures. Cartilage. (2018) 9:304–12. doi: 10.1177/1947603516686146 29156944 PMC6042030

[41] Grzanna MW Secor EJ Fortuno LV Au AY Frondoza CG. Anti-inflammatory effect of Carprofen is enhanced by avocado/soybean Unsaponifiables, glucosamine and chondroitin Sulfate combination in chondrocyte microcarrier spinner culture. Cartilage. (2020) 11:108–16. doi: 10.1177/1947603518783495 29938530 PMC6921959

[42] Teimourinejad A Hashemibeni B Salehi H Mostafavi F Kazemi M Bahramian H. Chondrogenic activity of two herbal products; pomegranate fruit extract and avocado/soybean unsaponifiable. Res Pharma Sci. (2020) 15:358. doi: 10.4103/1735-5362.293514, PMID: 33312214 PMC7714020

[43] Cake MA Read RA Guillou B Ghosh P. Modification of articular cartilage and subchondral bone pathology in an ovine meniscectomy model of osteoarthritis by avocado and soya unsaponifiables (ASU). Osteoarthr Cartil. (2000) 8:404–11. doi: 10.1053/joca.1999.0315, PMID: 11069724

[44] Kawcak CE Frisbie DD McIlwraith CW Werpy NM Park RD. Evaluation of avocado and soybean unsaponifiable extracts for treatment of horses with experimentally induced osteoarthritis. Am J Vet Res. (2007) 68:598–604. doi: 10.2460/ajvr.68.6.598, PMID: 17542691

[45] Boileau C Martel-Pelletier J Caron J Msika P Guillou GB Baudouin C. Protective effects of total fraction of avocado/soybean unsaponifiables on the structural changes in experimental dog osteoarthritis: inhibition of nitric oxide synthase and matrix metallopro- teinase-13. Arthritis Res Ther. (2009) 11:R41. doi: 10.1186/ar2649, PMID: 19291317 PMC2688188

[46] Altinel L Saritas ZK Kose KC Pamuk K Aksoy Y Serteser M. Treatment with unsaponifiable extracts of avocado and soybean increases TGF-beta1 and TGF-beta2 levels in canine joint fluid. Tohoku J Exp Med. (2007) 211:181–6. doi: 10.1620/tjem.211.181 17287602

[47] Appelboom T Schuerman J. Symptoms modifying effect of avocado/soybean unsaponifia- bles (ASU) in knee osteoarthritis. Scand J Rheumatol. (2001) 30:242–7. doi: 10.1080/030097401316909602 11578021

[48] Pavelka K Coste P Géher P Krejci G. Efficacy and safety of piascledine 300 versus chondroitin sulfate in a 6 months treatment plus 2 months observation in patients with osteoarthritis of the knee. Clin Rheumatol. (2010) 29:659–70. doi: 10.1007/s10067-010-1384-8 20179981

[49] Lequesne M Maheu E Cadet C Dreiser RL. Structural effect of avocado/soybean unsaponifiables on joint space loss in osteoarthritis of the hip. Arthritis Rheum. (2002) 47:50–8. doi: 10.1002/art1.10239 11932878

[50] Kwananocha I Vijarnsorn M Kashemsant N Lekcharoensuk C. Effectiveness of disease modifying osteoarthritis agents and carprofen for treatment of canine osteoarthritis. Thai. J Vet Med. (2016) 46:363–371. doi: 10.56808/2985-1130.2750

